# Association between triglyceride-glucose index and all-cause mortality in critically ill patients with ischemic stroke: analysis of the MIMIC-IV database

**DOI:** 10.1186/s12933-023-01864-x

**Published:** 2023-06-13

**Authors:** Weimin Cai, Jun Xu, Xiao Wu, Zhuoyan Chen, Liuwei Zeng, Xian Song, Yuan Zeng, Fujun Yu

**Affiliations:** 1 Department of Neurology, The First Affiliated Hospital of Wenzhou Medical University, Wenzhou, 325000 China; 2 Department of Gastroenterology and Hepatology, The First Affiliated Hospital of Wenzhou Medical University, No. 2, Fuxue Lane, Wenzhou, 325000 China

**Keywords:** Triglyceride-glucose index, Insulin resistance, Ischemic stroke, MIMIC-IV database, All-cause mortality

## Abstract

**Background:**

The triglyceride-glucose (TyG) index was significantly associated with insulin resistance (IR). Several studies have validated the effect of TyG index on cerebrovascular disease. However, the value of TyG index in patients with severe stroke requiring ICU admission remains unclear. The aim of this study was to investigate the association between the TyG index and clinical prognosis of critically ill patients with ischemic stroke (IS).

**Methods:**

This study identified patients with severe IS requiring ICU admission from the Medical Information Mart for Intensive Care (MIMIC-IV) database, and divided them into quartiles based on TyG index level. The outcomes included in-hospital mortality and ICU mortality. The association between the TyG index and clinical outcomes in critically ill patients with IS was elucidated using Cox proportional hazards regression analysis and restricted cubic splines.

**Results:**

A total of 733 patients (55.8% male) were enrolled. The hospital mortality and intensive care unit (ICU) mortality reached 19.0% and 14.9%, respectively. Multivariate Cox proportional hazards analysis showed that the elevated TyG index was significantly related to all-cause death. After confounders adjusting, patients with an elevated TyG index had a significant association with hospital mortality (adjusted hazard ratio, 1.371; 95% confidence interval, 1.053–1.784; P = 0.013) and ICU mortality (adjusted hazard ratio, 1.653; 95% confidence interval, 1.244–2.197; P = 0.001). Restricted cubic splines revealed that a progressively increasing risk of all-cause mortality was related to an elevated TyG index.

**Conclusion:**

The TyG index has a significant association with hospital and ICU all-cause death in critically ill patients with IS. This finding demonstrates that the TyG index might be useful in identifying patients with IS at high risk of all-cause death.

**Supplementary Information:**

The online version contains supplementary material available at 10.1186/s12933-023-01864-x.

## Introduction

Ischemic stroke (IS) occupied approximately 70% of stroke cases, and was the leading cause of disability and death disease globally [1]. Although endovascular therapy and intravenous t-PA have been used, the risk of adverse clinical outcomes in patients with IS still remains high, especially in critically ill patients [2, 3]. Several researchers have suggested that the incidence of IS is closely related to pathological and behavioral conditions, including diet, metabolic disorders, and smoking [4]. Various studies have also provided similar elucidations for associations between IS and metabolic disorders, such as hyperlipidemia and hyperglycemia [5, 6]. Published studies have demonstrated that triglyceride (TG) and diabetes are the main risk factors of cardiovascular disease (CAD) and cerebrovascular disease (CVD) [7, 8]. The triglyceride-glucose (TyG) index, consisting of fasting blood glucose (FBG) and TG, has become a convincing surrogate marker of insulin resistance (IR) [9, 10]. Furthermore, an increasing number of studies have reported that the TyG index is closely associated with increased poor cardiovascular events in the general population [11, 12], and in many other high-risk patient cohorts, such as stroke [13], diabetes [14] and hypertension [15].

TyG index provided an available approach to analyze the both of lipid metabolism and glucose status [16, 17]. Previous studies mostly concentrate on the level of TyG index in the general population to predict the adverse outcomes of CAD and CVD [11, 12]. Many scholars demonstrated that increased TyG index was strongly related to higher incidence of coronary atherosclerosis progression and death, which was verified by various coronary heart disease studies [18, 19]. In addition, several studies have also indicated the predictive power of the TyG index for the risk of stroke recurrence, morbidity, and even death in IS patients [20, 21]. Growing evidence have found that an elevated TyG index was significantly associated with higher risk of all-cause mortality [22, 23]. However, whether this association still existed in critically ill patients with IS, who usually had worse pathophysiological condition, remained unknown. Therefore, to assess whether the TyG index is a potential predicting tool for critically ill patients with IS may contribute to determining individuals at high risk of all-cause mortality for healthcare management or possible timely treatment.

Based on the current research status, the aim of this study was to assess the role of the TyG index in predicting all-cause mortality in critically ill patients with IS by analysing the Medical Information Mart for Intensive Care III (MIMIC-IV).

## Materials

### Study population

**Fig. 1:**
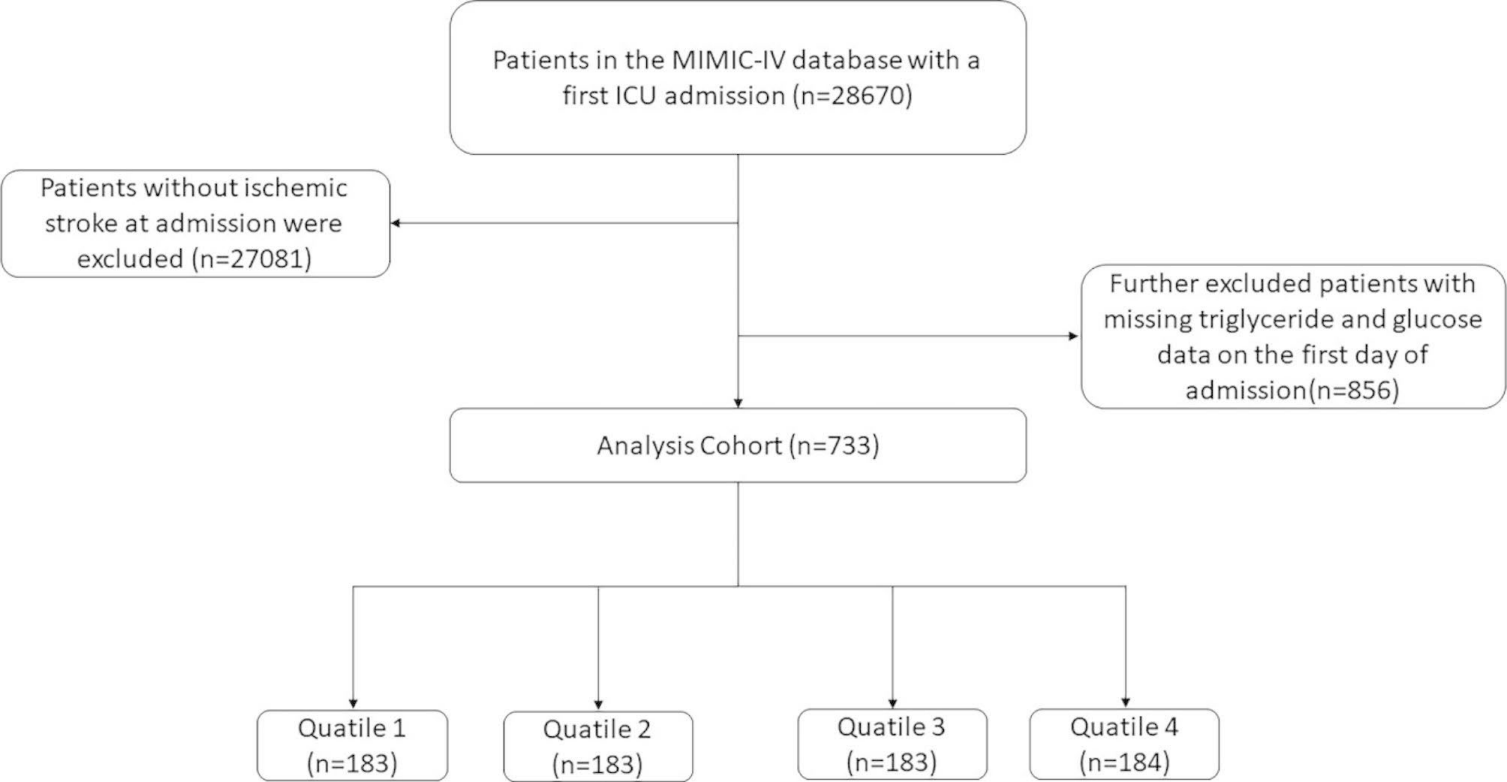
Flow of included patients through the trial

This retrospective study investigated health-related data obtained from the MIMIC-IV (version 2.2) database, which is a common and large database that was developed and managed by the MIT Computational Physiology Laboratory. This database is comprised of extensive and high-quality medical records of patients who were admitted to the intensive critical care units of the Beth Israel Deaconess Medical Center [24]. One author (Weimin Cai) complied with requirements for access to the database and was responsible for the data extraction. Patients who were diagnosed with the IS were enrolled in this study according to International Classification of Diseases, 9th and 10th Revision. The exclusion criteria were as follows: (1) patients aged less than 18 years at the time of first admission; (2) patients with multiple admissions to the ICU for IS, for whom only the first admission data were extracted; (3) patients with severe diseases such as end-stage renal dysfunction, cirrhosis, or cancer; (4) patients with an ICU length of stay of < 3 h. (5) patients without sufficient data (TG and FBG) on the first day of admission. Finally, a total of 773 patients were enrolled in this study and grouped into four groups based on the quartiles of the TyG index (Fig. 1).

### Data collection

The software PostgresSQL (version 13.7.2) and Navicate Premium (version 16) were used to extract information with a running Structured Query Language (SQL). The extraction of potential variables could be divided into four main groups: (1) demographics, such as age, gender, weight, height, and BMI. (2) comorbidities, including atrial fibrillation, sepsis, heart failure, diabetes, renal disease, and paraplegia. (3) laboratory indicators, including red blood cells (RBC), white blood cells (WBC), hemoglobin, platelet, serum sodium, serum creatinine, FBG and TG. (4) severity of illness scores at admission, including the Acute Physiology Score III (APSIII), the simplified Acute Physiology Score II (SAPS-II), Oxford Acute Severity of Illness Score (OASIS), and the Sepsis-related Organ Failure Assessment score (SOFA) [25, 26]. Follow-up began on the date of admission and ended on the date of death. The value of the TyG index was obtained as ln [fasting TG (mg/dl) × FBG (mg/dl)]/2 [21, 22]. All laboratory variables and disease severity scores were extracted from the data generated within the first 24 h after the patient entered the ICU.

To avoid possible bias, variables were excluded if they had more than 20% missing values. Variables with missing data less than 20% were processed by multiple imputation using a random forest algorithm (trained by other non-missing variables) by the “mice” package of R software (Additional file 1: Table S1) [27, 28].

### Clinical outcomes

The primary endpoint of the present study was hospital all-cause mortality, and the second endpoint was mortality in the ICU.

### Statistical analysis

Continuous variables were presented as mean ± SD or median (Interquartile range) according to data distribution, whereas categorical variables were expressed as proportions. The Kolmogorov-Smimov test was employed to evaluate the normality of continuous parameters. The analysis of continuous variables was performed using t-test or ANOVA if they presented a normal distribution and using Mann–Whitney U-test or Kruskal–Wallis test if they were non-normal distribution. Kaplan-Meier survival analysis was employed to assess the incidence rate of endpoints among groups based on different levels of the TyG index, and their differences were assessed through log-rank tests. To evaluate influencing factors related to the risk of all-cause death, binary logistic regression analysis was performed. Cox proportional hazards models were used to calculate the hazard ratio (HR) and 95% confidence interval (CI) between the TyG index and endpoints, and also adjusted for some models. Confounding variables included variables selected based on p value < 0.05 in univariate analysis. And clinically relevant and prognosis-associated variables were also enrolled in the multivariate model: model 1: unadjusted; model 2: adjusted for age, sex, and BMI; model 3: adjusted for age, sex, BMI, heart failure, atrial fibrillation, diabetes, sepsis, WBC, RBC, platelet, and serum creatinine. Further, we also analyzed the nonlinear association between baseline TyG index with ICU and in-hospital all-cause mortality using a restricted cubic spline regression model with four knots. The receiver operating characteristic (ROC) curves were analyzed to determine the cutoff value of the TyG index. The TyG index was entered into the models as continuous variables or ordinal variables (the first quartile of the TyG index was taken as a reference group). The P values for trends were calculated using the quartile level. Further stratified analyses were performed based on sex, age (≤ 65 and > 65 years), BMI (< 30 and ≥ 30 kg/m2), diabetes, heart failure, atrial fibrillation, and sepsis to identify the consistency of the prognostic value of the TyG index for primary outcomes. The interactions between TyG index and variables used for stratification were examined with likelihood ratio tests. A double-sided P < 0.05 was regarded as statistically significant. All statistical analysis was performed by the R software (version 4.0.2) and SPSS 22.0 (IBM SPSS Statistics, Armonk, NY, USA).

## Results

In this study, a total of 733 critically ill patients with IS were enrolled. The median age of the included patients was 69 (IQR: 58–79) years, and 409 (55.4%) were men. The median TyG index for all included participants was 8.90 (IQR:8.53–9.34). The hospital mortality and ICU mortality rates were 19.0% and 14.9%, respectively (Table 1).

**Table 1:** Characteristics and outcomes of participants categorized by TyG indexa^a^

Categories	Overall (N = 733 )	Q1 (N = 183)	Q2 (N = 183)	Q3 (N = 183)	Q4 (N = 184)	*χ2*/F	P-value
Age (years)	69 (58–79)	75 (61–84)	75 (63–81)	68 (58–78)	61 (50–69)	77.4	< 0.001
Height (cm)	168 (160–178)	168 (160–175)	168 (160–175)	170 (160–178)	172 (163–178)	12.65	0.005
Weight (kg)	79.3 (67.8–93.4)	74 (63.2–87.5)	74.8 (64.5–89.5)	80.4 (70-96.8)	87 (72.6-105.3)	49.56	< 0.001
BMI	27.5 (24.0-32.2)	26.0 (22.5–29.4)	26.8 (23.0-31.1)	27.8 (24.3–32.5)	29.9 (26.0-35.2)	44.50	< 0.001
Sex: male	409 (55.8)	97 (53.0)	100 (54.6)	104 (56.8)	108 (58.7)	1.33	0.711
SOFA	4 (2–6)	4 (2–6)	4 (2–6)	4 (2–6)	5 (3–7)	10.17	0.016
APS III	41 (30–57)	38 (29–53)	42 (29–55)	42 (31–59)	42 (31–62)	8.72	0.033
SAPS II	35 (28–34)	35 (27–42)	36 (29–45)	35 (28–44)	35 (27–46)	3.23	0.357
OASIS	33 (27–39)	31 (26–38)	33 (28–39)	33 (28–40)	34 (28–40)	5.65	0.130
GCS	14 (11–15)	14 (12–15)	14 (11–15)	14 (11–15)	15 (11–15)	1.624	0.615
Commorbidities							
Heart failure	207 (28.2)	49 (26.8)	68 (37.2)	48 (26.2)	42 (22.8)	10.40	0.018
Respiratory failure	285 (38.9)	43 (23.5)	72 (39.3)	73 (39.9)	97 (52.7)	33.14	< 0.001
Arterial fibrillation	293 (40.0)	77 (42.1)	90 (49.2)	73 (39.9)	53 (28.8)	16.37	< 0.001
Diabetes	268 (36.6)	41 (22.4)	62 (33.9)	65 (35.5)	100 (54.3)	41.56	< 0.001
Paraplegia	323 (44.1)	91 (49.7)	84 (45.9)	74 (40.4)	74 (40.2)	4.71	0.196
Renal disease	127 (17.3)	31 (16.9)	32 (17.5)	27 (14.8)	37 (20.1)	1.86	0.609
Sepsis	112 (15.3)	17 (9.3)	26 (14.2)	26 (14.2)	43 (23.4)	14.70	0.003
CCI	6 (5–8)	7 (5–8)	7 (5–9)	6 (4–8)	6 (4–8)	16.33	< 0.001
Laboratory tests							
WBC, K/uL	9.9 (7.6–13.1)	8.9 (6.8–11.0)	10.0 (7.7–12.8)	10.3 (7.5–14.0)	10.8 (8.6–14.2)	31.23	< 0.001
RBC, m/uL	3.7 (3.2–4.3)	3.8 (3.3–4.3)	3.8 (3.2–4.2)	3.7 (3.1–4.3)	3.7 (3.1–4.3)	3.80	0.283
Platelet, K/uL	219 (166–289)	207 (163–281)	228 (182–296)	217 (163–287)	221.5 (161–302)	4.51	0.211
Hemoglobin, g/dL	11.1 (9.4–12.8)	11.2 (9.5–13)	11.3 (9.7–12.7)	11.0 (9.2–12.9)	10.7 (9.1–12.8)	3.19	0.363
Sodium, mEq/L	140 (137–143)	140 (137–142)	140 (138–143)	140 (137–143)	139 (137–144)	1.36	0.712
Serum creatinine	0.9 (0.7–1.3)	0.9 (0.7–1.2)	0.9 (0.7–1.2)	0.9 (0.7–1.3)	1.0 (0.7–1.4)	2.68	0.441
TG, mg/d	114 (84–167)	71 (58–85)	102 (87–119)	140 (113–164)	221 (169–292)	490.41	< 0.001
FBG, mg/dL	125 (104–153)	104 (92–124)	121 (105–140)	129 (110–158)	157 (125–216)	171.54	< 0.001
TyG index	8.90 (8.53–9.34)	8.26 (8.09–8.41)	8.74 (8.64–8.82)	9.09 (8.99–9.21)	9.76 (9.53–10.03)	686.25	< 0.001
IV-tPA	129 (17.59)	21 (11.48)	25 (13.66)	32 (17.49)	51 (27.71)	19.68	< 0.001
Mechanical thrombectomy	74 (10.10)	21 (11.48)	20 (10.93)	17 (9.29)	16 (8.70)	1.05	0.789
Events							
LOS ICU, days	5 (2–10)	3 (2–7)	4 (2–8)	5 (2–12)	6.5 (3–13)	33.47	< 0.001
LOS hospital, days	13 (7–21)	9 (5–15)	12 (8–20)	13 (7–22)	16 (9.5–27)	42.76	0.011
ICU mortality	109 (14.9)	22 (12.0)	17 (9.3)	29 (15.8)	41 (22.3)	13.79	0.003
Hospital mortality	139 (19.0)	26 (14.2)	31 (16.9)	33 (18.0)	49 (26.6)	10.32	0.016

^a^ TyG index: Q1 (7.29–8.53), Q2 (8.53–8.90), Q3 (8.90–9.34), Q4 (9.34–11.34)

Abbreviation: TyG index, triglyceride glucose index; BMI, body mass index; SOFA, sequential organ failure assessment; CCI, Charlson comorbidity index; APSIII, acute physiology score III; SAPSII, simplifed acute physiological score II; OASIS, oxford acute severity of illness score; GCS, Glasgow coma scale; WBC, white blood cell; RBC, red blood cell; TG, triglyceride; FBG, fasting blood glucose; IV-tPA, intravenous tissue plasminogen activator

### Baseline characteristics

Baseline characteristics of critically ill patients with IS divided according to the TyG index quartiles are shown in the Table 1. Enrolled individuals were grouped into four groups based on the hospital admission TyG index levels [quartile (Q) 1: 7.29–8.53; Q2: 8.53–8.90; Q3: 8.90–9.34; Q4: 9.34–11.34]. The median value of the TyG index of each quartile was 8.26 (IQR: 8.09–8.41), 8.74 (IQR: 8.64–8.82), 9.09 (IQR: 8.99–9.21), and 9.76 (IQR: 9.53–10.03), respectively. Patients in the highest quartile of TyG index generally had higher body mass index (BMI), higher severity of illness scores on admission, higher prevalence of diabetes and sepsis, and higher level of WBC compared to the lower group. Compared to individuals in the lower quartile of TyG index, those in the higher quartile had longer hospital length of stay (9 days vs. 12 days vs. 13 days vs. 16 days, P = 0.011) and ICU length of stay (3 days vs. 4 days vs. 5 days vs. 6.5 days, P < 0.001), and higher hospital mortality (14.2% vs. 16.9% vs. 18.0% vs. 26.6%, P = 0.016). Since Q4 group had a better association with all-cause mortality, we further compared the difference between Q4 and Q1-3. The analysis showed that different grouping approach yielded similar results (Additional File 2, Table S2).

Baseline characteristics difference between survivors and non-survivors during the hospital stay are shown in Table 2. Patients in the non-survivor group were more likely to be male, and have higher severity of illness scores, higher prevalence of sepsis, higher value of WBC, RBC, platelet, creatinine. The TyG index levels in the non-survivor group were significantly higher than those in the survivor group (9.05 vs. 8.88, P = 0.005). Figure S1 depicted that the distribution of the TyG index stratified by the mortality status of all-cause in-hospital death and ICU death, respectively (Additional File 3, Figure S1).

**Table 2:** Baseline characteristics of the Survivors and Non-survivors groups

Categories	Overall (N = 773)	Survivor (N = 594)	Non-survivor (N = 139)	P-value
Age (years)	69 (58–79)	68.5 (57–78)	70 (59–81)	0.179
Height (cm)	168 (160–178)	168 (163–178)	168 (160–175)	0.116
Weight (kg)	79.3 (67.8–93.4)	79.3 (68-93.9)	78.4 (67.0-92.2)	0.582
BMI	27.5 (24.0-32.2)	27.4 (24.1–32.0)	27.7 (23.4–32.6)	0.997
Sex: male	409 (55.8)	342	67	0.028
SOFA	4 (2–6)	4 (2–6)	5 (3–8)	< 0.001
APS III	41 (30–57)	40 (29–55)	47 (35–65)	< 0.001
SAPS II	35 (28–34)	34 (27–43)	41 (33–49)	< 0.001
OASIS	33 (27–39)	32 (27–38)	36 (30–43)	< 0.001
GCS	14 (11–15)	14 (11–15)	15 (10–15)	0.196
Commorbidities				
Heart failure	207 (28.2)	164	43	0.247
Arterial fibrillation	293 (40.0)	229	64	0.064
Diabetes	268 (36.6)	211	57	0.134
Paraplegia	323 (44.1)	265	58	0.301
Renal disease	127 (17.3)	106	21	0.263
Sepsis	112 (15.3)	77	35	< 0.001
CCI	6 (5–8)	6 (4–8)	7 (5–9)	0.021
Laboratory tests				
WBC, K/uL	9.9 (7.6–13.1)	9.6 (7.4–12.6)	11.7 (8.4–15.8)	< 0.001
RBC, m/uL	3.7 (3.2–4.3)	3.8 (3.2–4.3)	3.7 (3.0-4.2)	0.032
Platelet, K/uL	219 (166–289)	225 (169–298)	207 (142–263)	0.001
Hemoglobin, g/dL	11.1 (9.4–12.8)	11.1 (9.5–12.9)	10.8 (9.0-12.5)	0.054
Sodium, mEq/L	140 (137–143)	140 (137–142)	140 (137–143)	0.675
Serum creatinine	0.9 (0.7–1.3)	0.9 (0.7–1.3)	1.1 (0.7–1.4)	0.018
TG, mg/d	114 (84–167)	114 (85–165)	117 (80–168)	0.812
FBG, mg/dL	125 (104–153)	120 (102–149)	137 (117–173)	< 0.001
TyG index	8.90 (8.53–9.34)	8.88 (8.51–9.29)	9.05 (8.64–9.54)	0.005
IV-tPA	129 (17.6)	108 (18.1)	21 (15.2)	0.392
Mechanical thrombectomy	74 (10.1)	59 (9.9)	15 (10.8)	0.762

Abbreviation: TyG index, triglyceride glucose index; BMI, body mass index; SOFA, sequential organ failure assessment; CCI, Charlson comorbidity index; APSIII, acute physiology score III; SAPSII, simplifed acute physiological score II; OASIS, oxford acute severity of illness score; GCS, Glasgow coma scale; WBC, white blood cell; RBC, red blood cell; TG, triglyceride; FBG, fasting blood glucose; IV-tPA, intravenous tissue plasminogen activator

### Primary outcomes

**Fig. 2:**
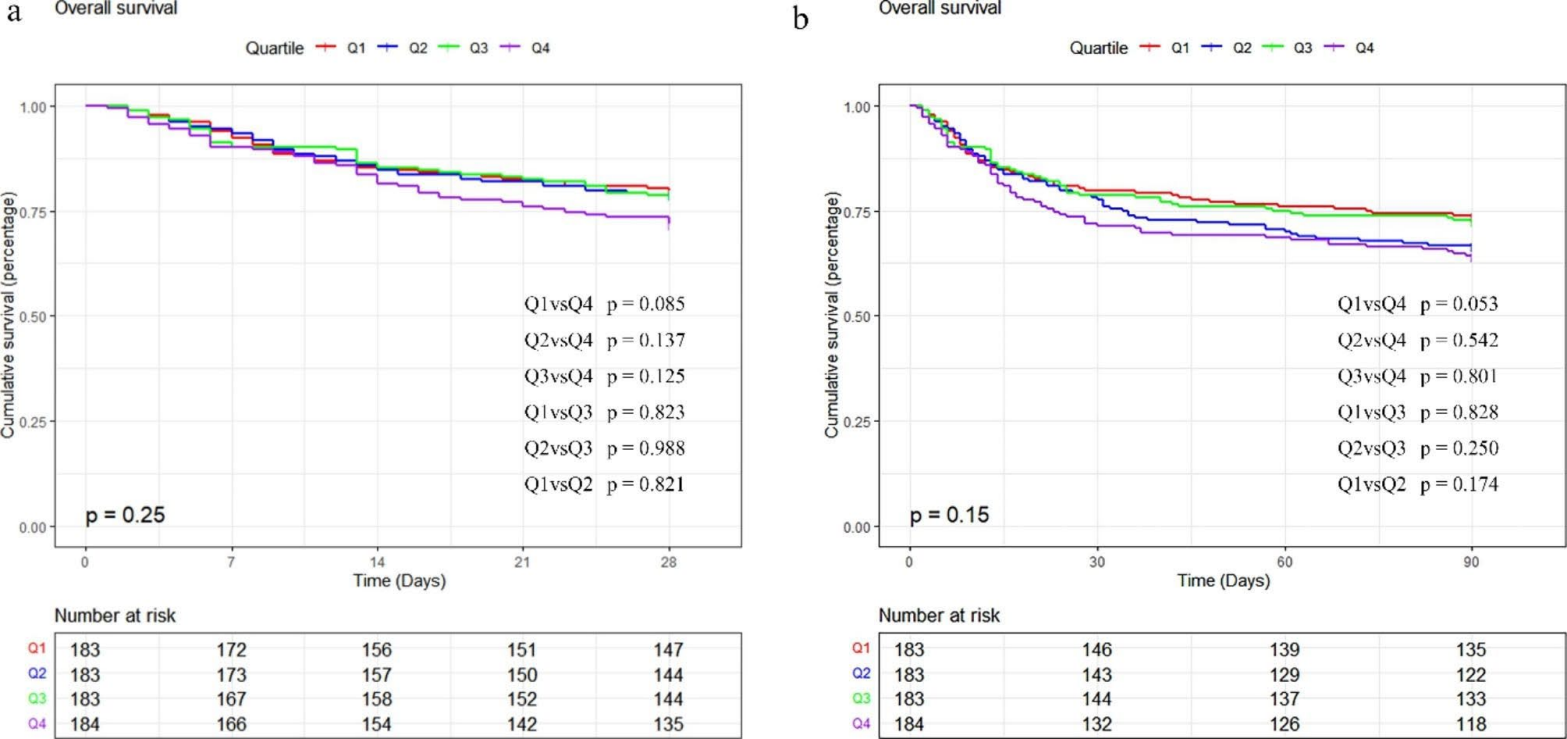
Kaplan–Meier survival analysis curves for all-cause mortality. Footnote TyG index quartiles: Q1 (7.29–8.53), Q2 (8.53–8.90), Q3 (8.90–9.34), Q4 (9.34–11.34). Kaplan–Meier curves showing cumulative probability of all-cause mortality according to groups at 28 days **(a)**, and 3 months **(b)**

The Kaplan-Meier survival analysis curves were employed to analyze incidence of primary outcomes among groups, based on the TyG index quartiles are presented in Fig. 2. Patients with a higher TyG index had a higher risk of hospital and ICU death. However, there was no significant difference during the short-term of 28 days and 3 months (log-rank P = 0.25, 0.15, respectively). We evaluated the TyG index clinical efficacy using the ROC analysis. However, the the AUC of TyG index was not good enough (in hospital death AUC:0.577, p = 0.004; ICU death AUC: 0.590, p = 0.004 ). The cutoff value of TyG index was 9.13 and 9.17 for hospital death and ICU death, respectively.

**Fig. 3:**
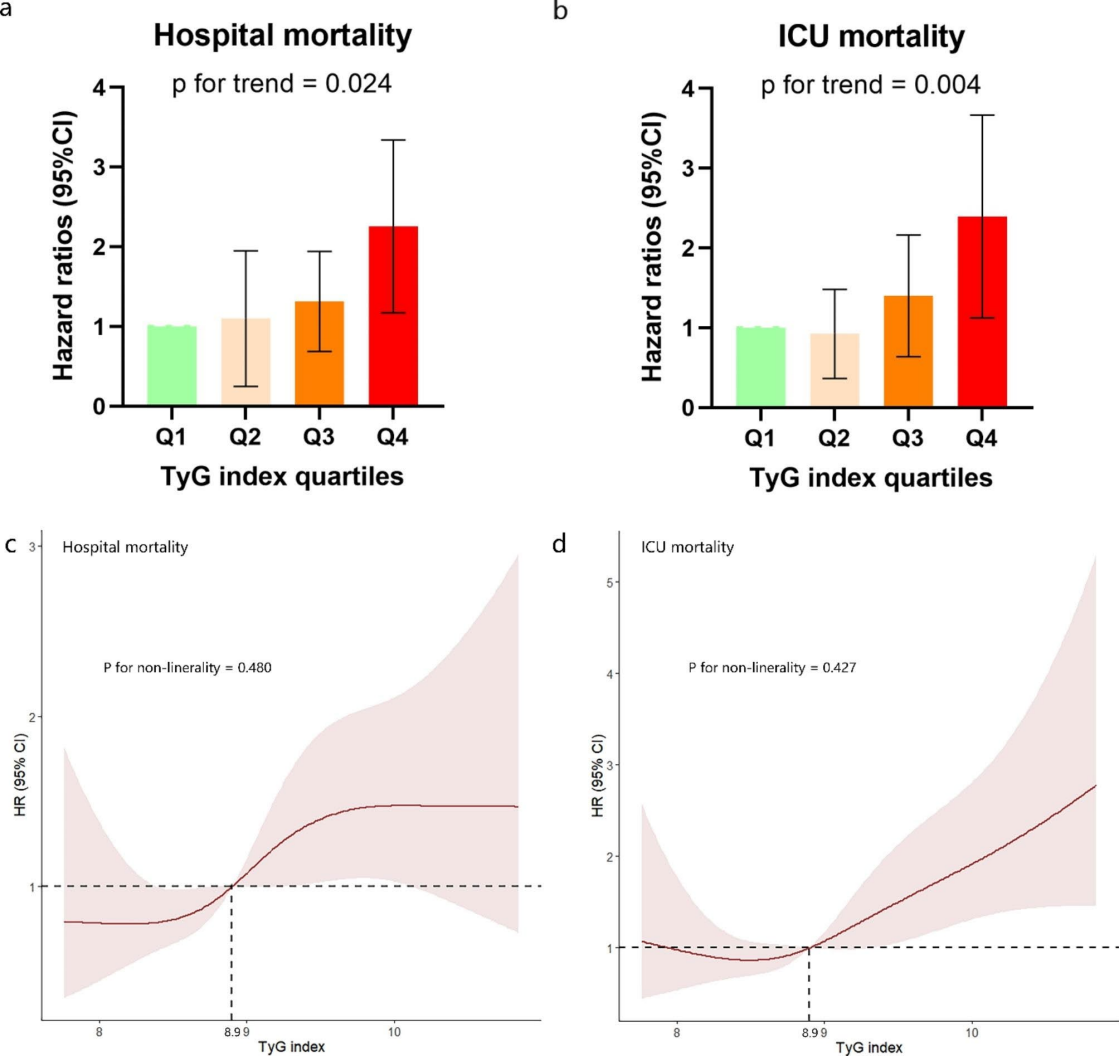
**(a), (b)**: Hazard ratios (95% CIs) for hospital mortality according to TyG index quartiles after adjusting for age, sex, BMI, heart failure, atrial fibrillation, diabetes, sepsis, white blood cell, red blood cell, platelet, serum creatinine. Error bars indicate 95% CIs. The frst quartile is the reference. **(c), (d)**: Restricted cubic spline curve for the TyG index hazard ratio. Heavy central lines represent the estimated adjusted hazard ratios, with shaded ribbons denoting 95% confdence intervals. TyG index 8.9 was selected as the reference level represented by the vertical dotted lines. The horizontal dotted lines represent the hazard ratio of 1.0. **(c)** Restricted cubic spline for hospital mortality. **(d)** Restricted cubic spline for ICU mortality. HR, hazard ratio; CI, confdence interval; ICU, intensive care unit; TyG, triglyceride-glucose

Table S3 (Additional File 4, Table S3) showed the results of the binary logistic regression for risk of all-cause death in critically ill patients with IS and variables with univariate analysis (p < 0.05), and factors that may influence prognosis obtained from clinicians’ suggestion and clinical experience were used as independent variables for binary logistic regression analysis. Results showed sex, sepsis, platelet, white blood cell, and TyG index were influential factors. Cox proportional risk analysis was used to analyze the association between TyG index and hospital mortality. And results demonstrated that TyG index was a significant risk factor in the unadjusted model [HR, 1.354 (95% CI 1.073–1.708) P = 0.011], partly adjusted model [HR, 1.516 (1.184–1.941) P = 0.001]and fully adjusted model [HR, 1.371 (1.053–1.784) P = 0.013] when the TyG index was a continuous variable. When TyG index was a nominal variable, patients in the higher quartile of TyG was significantly associated with higher risk of hospital death in the three established Cox proportional hazards models: unadjusted model [HR, 1.935 (95% CI 1.203–3.113) P = 0.007], partly adjusted model [HR, 2.367 (95% CI 1.441–3.888) P = 0.001] and fully adjusted model [HR, 2.026 (95% CI 1.215–3.377) P = 0.007], compared to subjects in the lowest quartile, and represented a tendency to increase with the TyG index (Table 3; Fig. 3a). Similar results were observed in the multivariate Cox proportional risk analysis of the TyG index and ICU mortality (Table 3; Fig. 3b). Furthermore, the restricted cubic splines regression model was used to reveal the risk of hospital mortality and ICU mortality increased linearly with increasing TyG index (P for non-linearity = 0.480 and P for non-linearity = 0.427, respectively) (Fig. 3c and d).

**Table 3:** Cox proportional hazard ratios (HR) for all-cause mortality

Categories	Model1			Model2			Model3		
	HR (95% CI)	P-value	P for trend	HR (95% CI)	P-value	P for trend	HR (95% CI)	P-value	P for trend
Hospital mortality									
Continuous variable per unit	1.354 (1.073–1.708)	0.011		1.516 (1.184–1.941)	0.001		1.371 (1.053–1.784)	0.013	
Quartile^a^			0.026			0.003			0.024
Q1 (N = 183)	Ref								
Q2 (N = 183)	1.200 (0.713–2.021)	0.493		1.218 (0.723–2.051)	0.459		1.218 (0.722–2.054)	0.459	
Q3 (N = 183)	1.268 (0.758–2.120)	0.365		1.385 (0.825–2.324)	0.218		1.158 (0.682–1.965)	0.587	
Q4 (N = 184)	1.935 (1.203–3.113)	0.006		2.367 (1.441–3.888)	0.001		2.026 (1.215–3.377)	0.007	
ICU mortality									
Continuous variable per unit	1.524 (1.187–1.957)	0.001		1.717 (1.312–2.248)	< 0.001		1.653 (1.244–2.197)	0.001	
Quartile^a^			0.007			0.001			0.004
Q1 (N = 183)	Ref								
Q2 (N = 183)	0.775 (0.411–1.458)	0.429		0.786 (0.417–1.479)	0.455		0.811(0.431–1.528)	0.516	
Q3 (N = 183)	1.313 (0.754–2.285)	0.336		1.427 (0.817–2.495)	0.212		1.262 (0.716–2.224)	0.421	
Q4 (N = 184)	1.914 (1.140–3.212)	0.014		2.307 (1.342–3.966)	0.002		2.172 (1.252–3.765)	0.006	

Model 1: unadjusted

Model 2: adjusted for age, sex, BMI

Model 3: adjusted for age, sex, BMI, heart failure, atrial fibrillation, diabetes, sepsis, IV-tPA, mechanical thrombectomy, white blood cell, red blood cell, platelet, serum creatinine

a TyG index: Q1 (7.29–8.53), Q2 (8.53–8.90), Q3 (8.90–9.34), Q4 (9.34–11.34)

### Subgroup analysis

**Fig. 4:**
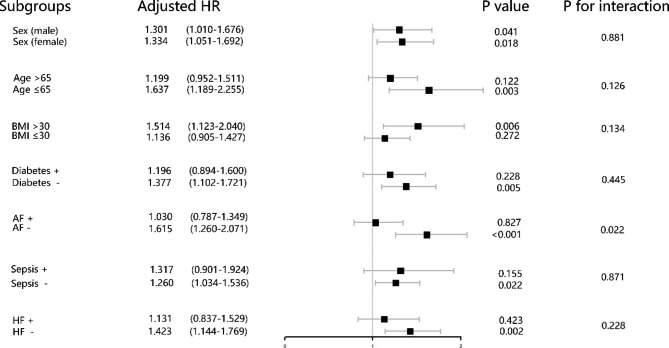
Forest plots of hazard ratios for the hospital mortality in different subgroups. HR, hazard ratio; CI, confidence interval; BMI, body mass index; AF, atrial fibrillation; HF, heart failure

**Fig. 5:**
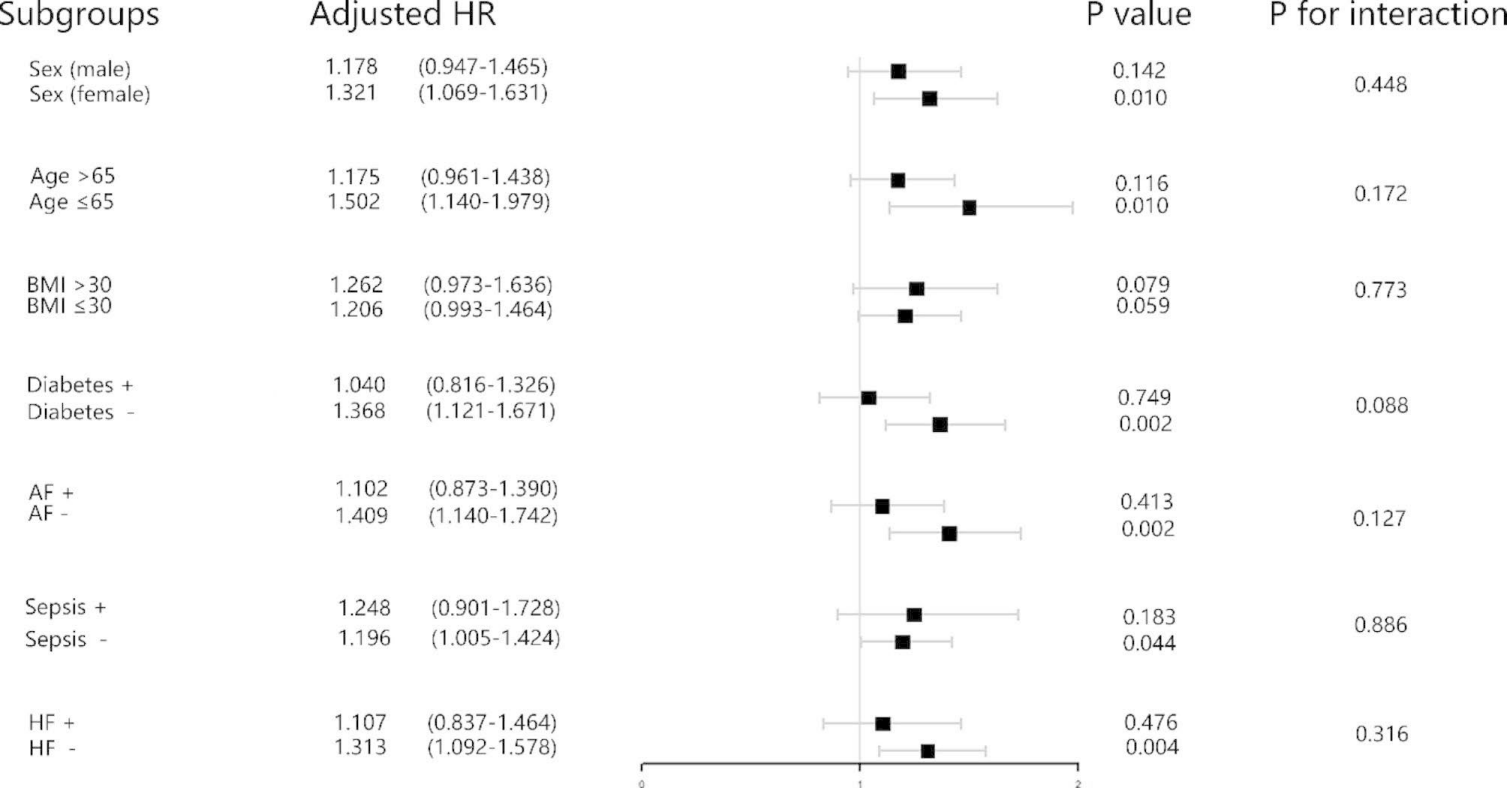
Forest plots of hazard ratios for the ICU mortality in different subgroups. HR, hazard ratio; CI, confidence interval; BMI, body mass index; AF, atrial fibrillation; HF, heart failure

The risk stratification value of the TyG index for primary endpoints was further analyzed in multiple subgroups of the enrolled patients, including sex, age, BMI, diabetes, atrial fibrillation, heart failure, and sepsis (Figs. 4 and 5). The TyG index was significantly associated with higher risk of hospital mortality in IS patients subgroups of female [HR (95% CI) 1.319 (1.068–1.628)], those aged < 65 years [HR (95% CI) 1.481 (1.126–1.950)], those with BMI < 30 kg/m^2^ [HR (95% CI) 1.214 (1.000-1.474)], those without diabetes [HR (95% CI) 1.368 (1.120–1.670)], those without atrial fibrillation [HR (95% CI) 1.413 (1.135–1.735)], those without sepsis [HR (95% CI) 1.200 (1.008–1.429)], those without heart failure [HR (95% CI) 1.312 (1.092–1.576)] (Fig. 4). Similarly, in terms of stratified analyses of ICU mortality, TyG index demonstrated significant association with higher risk of ICU mortality in subgroups of both of female [HR (95% CI) 1.327 (1.047–1.682)], and male [HR (95% CI) 1.306 (1.014–1.681)], those aged < 65 years [HR (95% CI) 1.609 (1.170–2.213)], those with BMI > 30 kg/m^2^ [HR (95% CI) 1.486 (1.105–1.998)], those without diabetes [HR (95% CI) 1.374 (1.110–1.717)], those without atrial fibrillation [HR (95% CI) 1.602 (1.250–2.053)], those without sepsis [HR (95% CI) 1.265 (1.038–1.540)], those without heart failure [HR (95% CI) 1.419 (1.142–1.763)]. Interestingly, the TyG index seemed to have more prominence of its predictive value in patients without atrial fibrillation [HR (95% CI) without atrial fibrillation 1.602 (1.250–2.053) vs. with atrial fibrillation 1.043 (0.798–1.363), P for interaction = 0.022] (Table 3; Fig. 5).

## Discussion

In the present study, we assessed the association between the TyG index and clinical outcomes in critically ill population with IS from a United States (US) cohort. The results of this study indicated that a higher TyG index had associations with all-cause ICU and hospital mortality in critically ill patients with IS. Even after adjustment for the confounding risk factors, the TyG index was still strongly associated with all-cause ICU and hospital mortality. Thus, the TyG index might be a promising decision-making tool for clinicians and may be an independent risk factor in critically ill patients with IS.

The TyG index, consisting of TG and FBG, has been suggested as a potential indicator of metabolic disorders, atherosclerotic disease, and cardiovascular disease [22, 29, 30]. Several clinical studies have investigated the relationship between the TyG index and cardiovascular disease morbidity and mortality in the general population or other patient populations. Yang et al. reported that increased TyG index was associated with a higher risk of neurologic worsening and mortality [31]. Lee et al. found that the TyG index may contribute to predicting the short-term functional outcome in patients with acute IS who received reperfusion therapy [32]. Liu et al. revealed the predictive effect of TyG index on clinical outcomes in acute IS patients with diabetes mellitus [30]. And for patients with coronary artery disease, the TyG index may be a promising predictor for future cardiovascular events in patients with coronary artery disease [33]. Another study that involving 5695 participants has suggested that monitoring the changes in the TyG index may play a role in predicting adverse cardiovascular events [34]. These studies indicated TyG held promise to predict clinical outcomes of cerebrovascular and cardiovascular related diseases.

However, several researchers have questioned the clinical efficacy of the TyG index for prognosis in IS patients [35, 36]. Firstly, the TyG index may be influenced by hyperglycemia and hyperlipidemia. Thus, the TyG index might not be applied in cerebrovascular disease patients if patients have extremely high TG and glucose levels [37]. In addition, compared to the TyG index, TG and FBG were more intuitive. And the TyG index may not reveal the longitudinal link between itself and cerebrovascular risk [35]. Thirdly, most of studies about the TyG index focused on elderly patients, and the predictive value of the TyG index is uncertainty in young individuals [38]. Some studies have offered the convincing answer to the above concerns. Insulin resistance (IR) is proven to be associated with several symptoms of metabolic syndrome including hyperlipidemia, obesity and hypertension. The HOMA-IR index, which has been widely employed to detect the function of β-cell and insulin resistance, is also limited in patients who have received insulin therapy or without functioning β-cells [39]. To demonstrate its clinical efficacy, the TyG index has been applied to evaluate IR in high-risk patients in large clinical trials. Guerroro et al. found that the TyG index can be an optimal method to assess the insulin resistance (IR) and had a high predictive power in young and middle-aged patients [9]. David et al. suggested that the TyG index seemed to be a better indicator than FBG and TG of the diagnosis of type II diabetes [40]. In other words, in certain situations, the TyG index is superior to single glucose or triglyceride and better reflects the development of the disease. Another study by Laura et al. revealed that the TyG index was closely associated with adverse cardiovascular events including cerebrovascular disease, peripheral arterial disease, and coronary heart failure, even independent of confounding risk factors [11]. Therefore, the TyG has been proven to be a strong hallmark of various cardiovascular, cerebrovascular diseases, and other metabolic-related diseases.

The exact biological mechanism underlying the relationship of the TyG index with the development and progression of cerebrovascular disease and mortality remains uncertain. The possible pathway may be related to insulin resistance. A previous study revealed that glucose may reflect IR from the liver, whereas TG reflects IR from adipose tissue. Thus, the TyG index may be strongly associated with IR from two aspects [41]. The TyG index is a biomarker of IR, which caused endothelium impairment, and inflammatory responses, accelerated the formation of foam cells, and promoted smooth muscle cell proliferation in the initiation of atherosclerosis [42–44]. Miao et al. reported that the TyG index was related to the degree of carotid atherosclerosis in patients with IR, and held promise to be atherosclerotic marker [45]. Ahn et al. suggested that the TyG index, calculated by lipid-related and glucose-related predictors, may become a creditable marker of IR [46]. Che et al. also indicated that a higher TyG index was associated with an elevated risk of cardiovascular disease after adjustment for the established confounding factors [47]. Several studies explained that IR is not only in the development of atherogenesis but also in advanced plaque progression through triggering apoptosis of vascular smooth muscle cells. Firstly, the TyG index could represent the situation of glucose metabolism, inflammatory responses, and oxidative stress. Secondly, the TyG index may reflect the metabolism of glycosylation products, and platelet reactivity, which can result in endothelial cell-dependent vasodilation [48]. Finally, an increased TyG index may elevate the level of free fatty acids, which may accompany IR. Lowering the TyG index seems to be an additional goal in patients at high risk of cerebrovascular disease [49–51]. All these physiological changes can contribute to the initiation and development of cerebrovascular diseases, and leading to poor clinical outcomes.

Current literature on the relationship between the TyG index and critically ill patients is few. Zhai et al. and Zhang et al. indicated that TyG index was strongly associated with in-hospital mortality in critically ill patients with heart disease [22, 52]. Zhang et al. found that the TyG index was associated with mortality in critically ill patients with stroke (including hemorrhage stroke and IS) [53]. However, in our specific cohort of ICU patients with IS, results showed the TyG index was a significant independent risk factor of greater mortality in critically ill patients. Additionally, for IS, a prevalent disease with high morbidity and mortality, we found that the TyG index could identify patients with high risk, which plays a role in clinical healthcare to reduce future major adverse outcomes.

In addition, this study further analyzed the risk stratification of various subgroups. Our subgroup analysis suggested that the value of the TyG index in predicting ICU mortality was consistent in male and female patients. However, we did not find any link between the TyG index and in-hospital all-cause mortality in included patients with diabetes, sepsis, atrial fibrillation, or heart failure at baseline. The reasons accounted for this phenomenon might be reverse causality: patients who have been diagnosed with these comorbidities were more likely to have accepted appropriate treatment or adopted healthy lifestyle habits. Therefore, their prognosis might be improved despite their high risk of all-cause mortality [11]. Moreover, the current study revealed that the predictive value of IR, evaluated by the TyG index seemed to be more prominent in patients without atrial fibrillation [HR (95% CI) without atrial fibrillation 1.602 (1.250–2.053) vs. with atrial fibrillation 1.043 (0.798–1.363), P for interaction = 0.022], indicating that atrial fibrillation treatment may have an important effect on the predictive performance of TyG index for all-cause mortality. The reason for this inconsistency might be atrial fibrillation patients are more likely to have accepted anticoagulant therapy before stroke. Previous studies have revealed that atrial fibrillation with earlier appropriate anticoagulation may decrease stroke mortality [54, 55]. Another interesting finding of the present study was that patients with higher TyG index were younger, and the association between TyG index and all-cause mortality seemed to be more significant in younger patients, which was also mentioned by the previous study [22]. Contrary to popular wisdom, clinicians may pay more attention to older patients because they may have more comorbidities, whereas our study calls for the same attention to be given to younger patients because they may have a higher mortality rate. In the present study, we also found a significant linear relationship between the TyG index and in-hospital mortality, indicating that the TyG index may be an accessible tool for detecting a high risk of mortality in critically ill patients with IS. Thus, maintaining optimal TG and glucose levels and taking better management of the TyG index play a role in reducing future major adverse clinical outcomes. In summary, the results of our analysis showed that the TyG index should not be regarded as a sole diagnostic tool, but should be used as a substitute along with other clinical and laboratory parameters to provide a more comprehensive assessment of an individual’s metabolic health and risk stratification of developing clinical outcomes such as post-stroke mortality during the clinical practice.

The major strength of this study was that we verified that elevated TyG index was an important independent risk factor of higher mortality in critically ill patients with IS in a US cohort. However, our study also had several limitations. First of all, due to its nature of retrospective design, this study could not definitively establish causality. Although multivariate adjustment and subgroup analyses were used, residual confounding factors could still have influenced the clinical outcomes. The potential confounders, such as IS subtypes, National Institutes of Health Stroke Scale elimination, time of stroke, and cause of death was unobtainable in this database. Secondly, only the baseline TyG index was analyzed in this study. Dynamic changes of the TyG index were unavailable during the hospital and ICU stay. Therefore, the predictive power of the TyG index change is also needed to be evaluated in future research. Thirdly, our study did not perform the hyperinsulinemic-euglycemic clamp test, so we cannot evaluate the association between the TyG index and the gold standard of insulin resistance.

## Conclusions

In summary, our results extended the utility of the TyG index to critically ill patients with IS and demonstrated that the TyG index could be used as a potential index for risk stratification of in-hospital and ICU mortality among these patients. Monitoring the TyG index could contribute to decision-making and disease management in clinical practice. Further studies are needed to determine whether taking better control of the TyG index will improve clinical prognosis in the future.

## Electronic supplementary material

Below is the link to the electronic supplementary material.

Additional File 1. Table S1

Additional File 2. Table S2

Additional File 3. Figure S1

Additional File 4. Table S3

## Data Availability

The datasets generated and analyzed during the current study are available from the corresponding author on reasonable request.

## Abbreviations

IS: Ischemic stroke
ICU: Intensive care unit
IR: Insulin resistance
TyG: Triglyceride-glucose
MIMIC-IV: Medical Information Mart for Intensive Care
CAD: Cardiovascular disease
CVD: Cerebrovascular disease
TG: Triglyceride
FBG: Fasting blood glucose
SQL: Structured Query Language
APSIII: Acute Physiology Score III
SAPS-II: Simplified Acute Physiology Score II
OASIS Oxford: Acute Severity of Illness Score
SOFA: Sepsis-related Organ Failure Assessment score
CI: Confidence interval
HR: Hazard ratio
BMI: Body mass index
IQR: Interquartile range
WBC: White blood cell
RBC: Red blood cell
IV-Tpa: Intravenous tissue plasminogen activator
HOMA-IR: Homeostatic model assessment of IR
US: United states
