# Supplementary material for: Association between triglyceride-glucose index and all-cause mortality in critically ill patients with ischemic stroke: analysis of the MIMIC-IV database
Source: Cardiovasc Diabetol. 2023 Jun 13;22:138. doi: 10.1186/s12933-023-01864-x (PMC10262584; doi:10.1186/s12933-023-01864-x)
Supplement: Supplementary file 1 — Additional File 1. Table S1 [file 12933_2023_1864_MOESM1_ESM.docx]

**Table S1. missing number for risk variables and outcome variables**

| Risk Variables | Missing number (%) |
| --- | --- |
| Age (years) | 0 |
| Height (cm) | 5 (0.68%) |
| Weight (kg) | 7 (0.95%) |
| BMI | 13 (1.77%) |
| Sex: male | 0 |
| SOFA | 0 |
| APS III | 0 |
| SAPS II | 0 |
| OASIS | 0 |
| GCS | 0 |
| Commorbidities |  |
| Heart failure | 0 |
| Respiratory failure | 0 |
| Arterial fibrillation | 0 |
| Diabetes | 0 |
| Paraplegia | 0 |
| Renal disease | 0 |
| Sepsis | 0 |
| CCI | 0 |
| Laboratory tests |  |
| WBC, K/uL | 1 (0.14%) |
| RBC, m/uL | 4 (0.55%) |
| Platelet, K/uL | 4 (0.55%) |
| Hemoglobin, g/dL | 4 (0.55%) |
| Sodium, mEq/L | 0 |
| Serum creatinine | 0 |
| TG, mg/d | 0 |
| FBG, mg/dL | 0 |
| TyG index | 0 |
| IV-tPA | 0 |
| Mechanical thrombectomy | 0 |

Abbreviation: TyG index, triglyceride glucose index; BMI, body mass index; SOFA, sequential organ failure assessment; CCI, Charlson comorbidity index; APSIII, acute physiology score III; SAPSII, simplifed acute physiological score II; OASIS, oxford acute severity of illness score; GCS, Glasgow coma scale; WBC, white blood cell; RBC, red blood cell; TG, triglyceride; FBG, fasting blood glucose; IV-tPA, intravenous tissue plasminogen activator
