# Supplementary material for: Association between triglyceride-glucose index and all-cause mortality in critically ill patients with ischemic stroke: analysis of the MIMIC-IV database
Source: Cardiovasc Diabetol. 2023 Jun 13;22:138. doi: 10.1186/s12933-023-01864-x (PMC10262584; doi:10.1186/s12933-023-01864-x)
Supplement: Supplementary file 2 — Additional File 2. Table S2 [file 12933_2023_1864_MOESM2_ESM.docx]

| **Table S2.** Characteristics and outcomes of participants categorized by TyG index^a^ | | | | | | |
| --- | --- | --- | --- | --- | --- | --- |
| Categories | Overall (N=733 ) | Q1-Q3 (N=549) | | | Q4 (N=184) | P-value |
| Age (years) | 69 (58-79) | 72 (61-80) | | | 61 (50-69) | <0.001 |
| Height (cm) | 168 (160-178) | 168 (160-178) | | | 172 (163-178) | 0.012 |
| Weight (kg) | 79.3 (67.8-93.4) | 77.3 (65.7-90.6) | | | 87 (72.6-105.3) | <0.001 |
| BMI | 27.5 (24.0-32.2) | 27.1 (23.6-31.2) | | | 29.9 (26.0-35.2) | <0.001 |
| Sex: male | 409 (55.8) | 301 (54.8) | | | 108 (58.7) | 0.391 |
| SOFA | 4 (2-6) | 4 (2-6) | | | 5 (3-7) | 0.005 |
| APS III | 41 (30-57) | 41 (30-55) | | | 42 (31-62) | 0.094 |
| SAPS II | 35 (28-34) | 35 (28-43) | | | 35 (27-46) | 0.974 |
| OASIS | 33 (27-39) | 33 (27-39) | | | 34 (28-40) | 0.455 |
| GCS | 14 (11-15) | 14 (11-15) | | | 15 (11-15) | 0.181 |
| Commorbidities |  |  |  |  |  |  |
| Heart failure | 207 (28.2) | 165 (30.5) | | | 42 (22.8) | 0.072 |
| Respiratory failure | 285 (38.9) | 188 (34.2) | | | 97 (52.7) | <0.001 |
| Arterial fibrillation | 293 (40.0) | 240 (43.7) | | | 53 (28.8) | <0.001 |
| Diabetes | 268 (36.6) | 168 (30.6) | | | 100 (54.3) | <0.001 |
| Paraplegia | 323 (44.1) | 249 (45..) | | | 74 (40.2) | 0.231 |
| Renal disease | 127 (17.3) | 90 (16.4) | | | 37 (20.1) | 0.261 |
| Sepsis | 112 (15.3) | 69 (12.5) | | | 43 (23.4) | 0.001 |
| CCI | 6 (5-8) | 7 (5-8) | | | 6 (4-8) | <0.001 |
| Laboratory tests |  |  |  |  |  |  |
| WBC, K/uL | 9.9 (7.6-13.1) | 9.6 (7.4-12.6) | | | 10.8 (8.6-14.2) | 0.001 |
| RBC, m/uL | 3.7 (3.2-4.3) | 3.75 (3.22-4.28) | | | 3.7 (3.1-4.3) | 0.298 |
| Platelet, K/uL | 219 (166-289) | 218 (167-285) | | | 221.5 (161-302) | 0.922 |
| Hemoglobin, g/dL | 11.1 (9.4-12.8) | 11.2 (9.5-12.9) | | | 10.7 (9.1-12.8) | 0.147 |
| Sodium, mEq/L | 140 (137-143) | 140 (138-143) | | | 139 (137-144) | 0.302 |
| Serum creatinine | 0.9 (0.7-1.3) | 0.9 (0.7-1.3) | | | 1.0 (0.7-1.4) | 0.123 |
| TG, mg/d | 114 (84-167) | 99 (76-127) | | | 221 (169-292) | <0.001 |
| FBG, mg/dL | 125 (104-153) | 117 (101-139) | | | 157 (125-216) | <0.001 |
| TyG index | 8.90 (8.53-9.34) | 8.74 (8.41-8.99) | | | 9.76 (9.53-10.03) | <0.001 |
|  |  |  | | |  |  |
| IV-tPA | 129 (17.6) | 78 (16.6) | | | 51 (38.3) | <0.001 |
| Mechanical thrombectomy | 74 (10.1) | 58 (10.5) | | | 16 (9.5) | 0.532 |
| Events |  |  |  |  |  |  |
| LOS ICU, days | 5 (2-10) | 4 (2-9) | | | 6.5 (3-13) | <0.001 |
| LOS hospital, days | 13 (7-21) | 12 (6-20) | | | 16 (9.5-27) | <0.001 |
| ICU mortality | 109 (14.9) | 68 (12.4) | | | 41 (22.3) | 0.002 |
| Hospital mortality | 139 (19.0) | 90 (16.4) | | | 49 (26.6) | 0.003 |

^a^ TyG index: Q1-Q3 (7.29–9.34), Q4 (9.34–11.34)

Abbreviation: TyG index, triglyceride glucose index; BMI, body mass index; SOFA, sequential organ failure assessment; CCI, Charlson comorbidity index; APSIII, acute physiology score III; SAPSII, simplifed acute physiological score II; OASIS, oxford acute severity of illness score; GCS, Glasgow coma scale; WBC, white blood cell; RBC, red blood cell; TG, triglyceride; FBG, fasting blood glucose; IV-tPA, intravenous tissue plasminogen activator
