# Supplementary figures and images for: Association between triglyceride-glucose index and all-cause mortality in critically ill patients with ischemic stroke: analysis of the MIMIC-IV database
Source: Cardiovasc Diabetol. 2023 Jun 13;22:138. doi: 10.1186/s12933-023-01864-x (PMC10262584; doi:10.1186/s12933-023-01864-x)

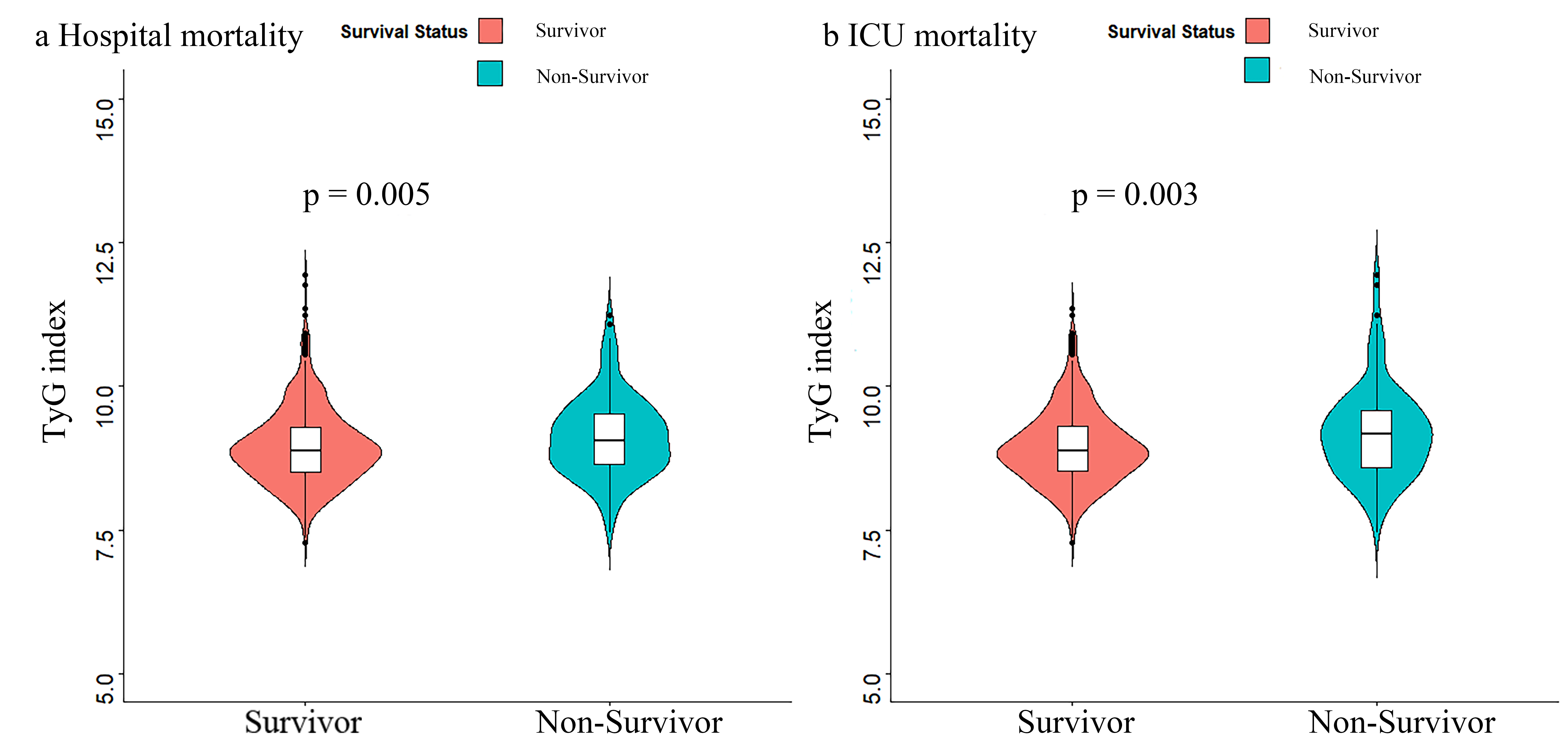

Supplement: Supplementary file 3 — Additional File 3. Figure S1 [file 12933_2023_1864_MOESM3_ESM.tif]
