# Supplementary material for: Association between triglyceride-glucose index and all-cause mortality in critically ill patients with ischemic stroke: analysis of the MIMIC-IV database
Source: Cardiovasc Diabetol. 2023 Jun 13;22:138. doi: 10.1186/s12933-023-01864-x (PMC10262584; doi:10.1186/s12933-023-01864-x)
Supplement: Supplementary file 4 — Additional File 4. Table S3 [file 12933_2023_1864_MOESM4_ESM.docx]

Table S3 Binary logistic regression analysis of the factors influencing all-cause death of the study population.

| Variables | OR | Lower 95% CI | Upper 95% CI | P-value |
| --- | --- | --- | --- | --- |
| Sex: male | 0.607 | 0.405 | 0.911 | 0.016 |
| Age | 1.014 | 0.998 | 1.029 | 0.079 |
| Serum creatinine | 1.034 | 0.873 | 1.224 | 0.700 |
| Platelet | 0.996 | 0.994 | 0.998 | 0.001 |
| TyG index | 1.400 | 1.017 | 1.929 | 0.039 |
| IV-tPA | 0.582 | 0.324 | 1.046 | 0.070 |
| Mechanical thrombectomy | 1.662 | 0.882 | 3.133 | 0.116 |
| RBC | 0.911 | 0.682 | 1.217 | 0.529 |
| Heart failure | 1.068 | 0.687 | 1.661 | 0.770 |
| Diabetes | 1.205 | 0.789 | 1.840 | 0.389 |
| Sepsis | 2.367 | 1.396 | 4.012 | 0.001 |
| WBC | 1.048 | 1.013 | 1.083 | 0.006 |

Abbreviation: TyG index, triglyceride glucose index; IV-tPA, intravenous tissue plasminogen activator; RBC, Red blood cell; WBC, white blood cell
